# Development of a Multivariate Prediction Model for Early-Onset Bronchiolitis Obliterans Syndrome and Restrictive Allograft Syndrome in Lung Transplantation

**DOI:** 10.3389/fmed.2017.00109

**Published:** 2017-07-17

**Authors:** Angela Koutsokera, Pierre J. Royer, Jean P. Antonietti, Andreas Fritz, Christian Benden, John D. Aubert, Adrien Tissot, Karine Botturi, Antoine Roux, Martine L. Reynaud-Gaubert, Romain Kessler, Claire Dromer, Sacha Mussot, Hervé Mal, Jean-François Mornex, Romain Guillemain, Christiane Knoop, Marcel Dahan, Paola M. Soccal, Johanna Claustre, Edouard Sage, Carine Gomez, Antoine Magnan, Christophe Pison, Laurent P. Nicod, The SysCLAD Consortium

**Affiliations:** ^1^Division of Pulmonary Medicine, Centre Hospitalier Universitaire Vaudois (CHUV), University of Lausanne, Lausanne, Switzerland; ^2^Institut du thorax, INSERM UMR 1087/CNRS UMR 6291, CHU de Nantes, Université de Nantes, Nantes, France; ^3^Biomax Informatics AG, Planegg, Germany; ^4^Division of Pulmonary Medicine, University Hospital Zurich, Zurich, Switzerland; ^5^Pneumology, Adult CF Center and Lung transplantation Department, Foch Hospital, Université Versailles Saint-Quentin-en-Yvelines, UPRES EA220, Suresnes, France; ^6^Pulmonary Medicine, CF Center and Lung Transplantation Department, Centre Hospitalier Universitaire Nord, CNRS UMR 6236 Aix-Marseille Université, Marseille, France; ^7^Lung Transplant Center, Hôpitaux universitaires de Strasbourg, Strasbourg, France; ^8^Service des Maladies respiratoires, Hôpital Haut Lévèque, Pessac, France; ^9^Service de Chirurgie Thoracique, Vasculaire et Transplantation Cardiopulmonaire, Hôpital Marie Lannelongue, Le Plessis Robinson, France; ^10^Service de Pneumologie et Transplantation pulmonaire, Hôpital Bichat, Université Denis Diderot, INSERM UMR1152, Paris, France; ^11^Université de Lyon, INRA UMR 754, Hospices civils de Lyon, Lyon, France; ^12^Assistance Publique Hôpitaux de Paris, Paris, France; ^13^Department of Chest Medicine, Erasme University Hospital, Brussels, Belgium; ^14^CHU Larrey, Toulouse, France; ^15^Division of Pulmonary Medicine, Geneva University Hospitals, Geneva, Switzerland; ^16^Clinique Universitaire de Pneumologie, Pôle Thorax et Vaisseaux, CHU Grenoble, INSERM 1055, Université Grenoble Alpes, Grenoble, France; ^17^Thoracic Surgery Department, Foch Hospital, Université Versailles Saint-Quentin-en-Yvelines, UPRES EA220, Suresnes, France; ^18^Full List of the Authors Is Given in the Section SysCLAD Consortium Members

**Keywords:** chronic lung allograft dysfunction, bronchiolitis obliterans syndrome, restrictive allograft syndrome, chronic rejection, predictive model

## Abstract

**Background:**

Chronic lung allograft dysfunction and its main phenotypes, bronchiolitis obliterans syndrome (BOS) and restrictive allograft syndrome (RAS), are major causes of mortality after lung transplantation (LT). RAS and early-onset BOS, developing within 3 years after LT, are associated with particularly inferior clinical outcomes. Prediction models for early-onset BOS and RAS have not been previously described.

**Methods:**

LT recipients of the French and Swiss transplant cohorts were eligible for inclusion in the SysCLAD cohort if they were alive with at least 2 years of follow-up but less than 3 years, or if they died or were retransplanted at any time less than 3 years. These patients were assessed for early-onset BOS, RAS, or stable allograft function by an adjudication committee. Baseline characteristics, data on surgery, immunosuppression, and year-1 follow-up were collected. Prediction models for BOS and RAS were developed using multivariate logistic regression and multivariate multinomial analysis.

**Results:**

Among patients fulfilling the eligibility criteria, we identified 149 stable, 51 BOS, and 30 RAS subjects. The best prediction model for early-onset BOS and RAS included the underlying diagnosis, induction treatment, immunosuppression, and year-1 class II donor-specific antibodies (DSAs). Within this model, class II DSAs were associated with BOS and RAS, whereas pre-LT diagnoses of interstitial lung disease and chronic obstructive pulmonary disease were associated with RAS.

**Conclusion:**

Although these findings need further validation, results indicate that specific baseline and year-1 parameters may serve as predictors of BOS or RAS by 3 years post-LT. Their identification may allow intervention or guide risk stratification, aiming for an individualized patient management approach.

## Introduction

Chronic lung allograft dysfunction (CLAD) is the principal cause of poor long-term survival in lung transplantation (LT) and, although no international consensus definition has been developed to date, CLAD refers to the persistent decline of the forced expiratory volume in one second (FEV_1_) that cannot be attributed to a specific cause other than chronic graft rejection (1, 2). Bronchiolitis obliterans syndrome (BOS) and restrictive allograft syndrome (RAS) are considered to be the two main phenotypes of CLAD (2, 3). BOS is characterized by persistent airflow obstruction in the absence of a restrictive ventilation defect and imaging studies that may be unremarkable or show air trapping (4). Early-onset BOS, developing within 2 or 3 years after LT, is associated with particularly unfavorable outcomes, resulting in high morbidity and mortality soon after LT (5, 6). For RAS, various diagnostic criteria have been used in different studies, but overall, RAS is characterized by a restrictive ventilation defect and radiological signs of fibrosis or infiltrates (7–9). Prognosis is worse for RAS than BOS, and for RAS, time of diagnosis after transplant does not seem to influence survival (10). Diagnosis of BOS or RAS requires exclusion of alternative diagnoses, and this may be challenging (1).

Although the pathogenic mechanisms and the risk factors implicated in BOS and RAS are not fully elucidated (4, 11), recent literature provides increasing evidence and novel insights. Concerning BOS, most studies emphasize the role of multiple allo-immune and non-immune mechanisms (4, 11–13), but information on the risk factors of RAS or a comparative analysis of RAS and BOS is limited (10, 14). Identification of clinical risk factors associated with specific CLAD phenotypes is of particular clinical importance as it may assist patient risk stratification, optimize follow-up, or allow early intervention for potentially modifiable factors.

Multivariate prediction models are being increasingly developed to help health-care providers estimate the probability of a future disease (15). A few studies described clinical predictive models for BOS (16–19), but to our knowledge, no study has used this approach for BOS and RAS. The SysCLAD (systems prediction of CLAD) study is a collaborative project merging data from two large European LT cohorts. It aims to develop prediction models for early-onset BOS and RAS by implementing a multilevel approach and incorporating multiple clinical and laboratory parameters (20). The present study concerns the clinical arm of SysCLAD and describes the development of a multivariate predictive model for early-onset BOS and RAS. Our methodology and findings are reported in accordance with the TRIPOD recommendations (15). Some of the results of this study have been previously reported as conference abstracts (21, 22).

## Materials and Methods

### Patient Population

This multicenter cohort study used data from two prospective European LT cohorts, the French–Belgian Cohort of LT (COLT) and the Swiss Transplant Cohort Study (STCS). COLT includes 11 French centers (Bordeaux, Grenoble, Lyon, Marseille, Nantes, Georges Pompidou Hospital, Hospital Bichat in Paris, Centre Chirurgical Marie Lennelongue in Le Plessis-Robinson, Strasbourg, Toulouse, Foch Hospital in Suresnes) and the Erasme center in Brussels, Belgium. It was established in October 2009. STCS is a multicenter cohort collecting data from all Swiss transplant programs (23, 24). The two Swiss LT centers, Zurich and Lausanne/Geneva, participate in STCS since its establishment in April 2008.

COLT and STCS patients were eligible for the SysCLAD cohort if they were alive with at least 2 years of follow-up but less than 3 years, or if they died or were retransplanted at any time less than 3 years. Only patients with a first LT were included in the study. Data collection concerned the periods between October 2009 (for COLT) or April 2008 (for STCS) and February 2014 (for both cohorts). Data harmonization, completion, and quality control were performed during the following 1.5 years. The corresponding national and local ethics committees approved the study protocol, and all participants provided written informed consent.

### Assessment by the Adjudication Committee

Outcomes were established by an adjudication committee of LT specialists (Antoine Magnan, Christophe Pison, Antoine Roux, Martine L. Reynaud-Gaubert, Laurent P. Nicod, John D. Aubert, and Christian Benden) who evaluated pulmonary function tests (PFTs), imaging studies, and confounding factors. This committee formed a group opinion and identified LT recipients who remained stable or developed a definite BOS or RAS. Actions to blind assessment included data anonymization and an initial evaluation of the PFTs before assessment of imaging studies and confounding factors.

Bronchiolitis obliterans syndrome was defined as the persistent drop of FEV_1_ in the absence of a restrictive defect and in the absence of confounding factors (4, 8). RAS was defined as the persistent decline of FEV_1_ in the presence of a restrictive defect [i.e., decline of total lung capacity (TLC) and/or forced vital capacity (FVC)] and in the absence of confounding factors (8, 25). Imaging studies compatible with RAS were used as an additional diagnostic criterion. Patients were censored either at death/retransplantation or they were scored as uncensored at their last available assessment between 2 and 3 years of follow-up. More details on the elements assessed are presented below.

#### Pulmonary Function Tests

In addition to specific time-point values recorded in the databases, PFTs were retrieved from LT centers. Spirometry was performed at every patient visit. The frequency of TLC measurements varied among centers: Zurich conducted TLC measurements at each patient visit, whereas in other centers, this was done at predefined assessment visits and when clinically indicated.

All hospitals calibrated their spirometers and body plethysmographers routinely according to the ATS/ERS guidelines. Home spirometry measurements were not used for diagnostic purposes in the present study. All pediatric patients included were able to perform PFTs reliably.

#### Imaging Studies

Chest X-rays and chest CT scans of patients with declining PFTs were assessed to identify confounding factors for this decline or to support the PFT-based diagnosis of BOS or RAS. Unremarkable imaging studies or signs of air trapping (4) were considered supportive of BOS, whereas fibrosis, pleural thickening, or infiltrates in the absence of a confirmed infection were considered supportive of RAS (8, 25).

#### Confounding Factors

Before establishing the diagnosis of BOS or RAS, we excluded allograft or extra-allograft factors that could cause the decline of PFTs (1, 4). Anastomotic, parenchymal, or thoracic wall abnormalities were identified using imaging studies and bronchoscopy reports. Whenever necessary, LT centers were contacted to obtain additional information.

#### Establishment of the Diagnosis

**Figure 1:**
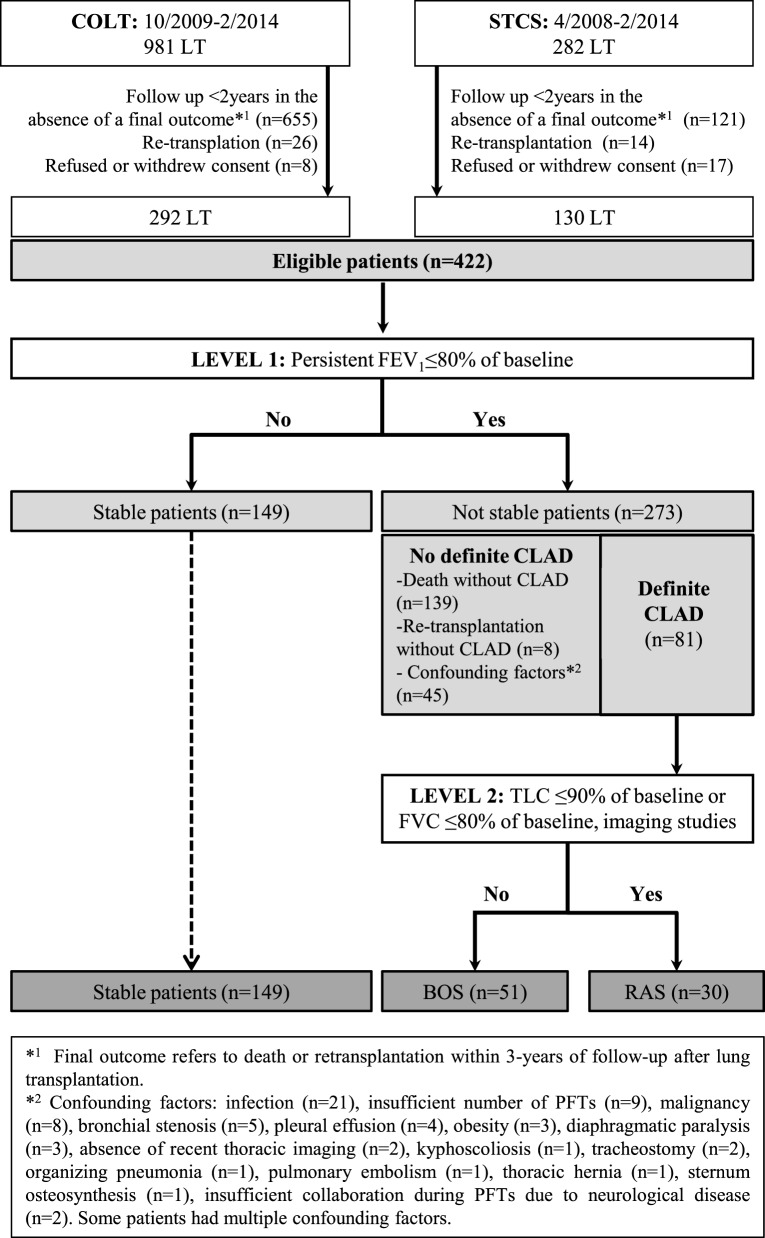
Flow chart diagram of the evaluated population.

A flow chart diagram of the assessed population is shown in Figure 1. During level-1 analysis, FEV_1_ baseline was calculated as the mean of the two best FEV_1_ measured 3 weeks apart. At the last assessment time-point, FEV_1_% of baseline was calculated to identify two groups of patients: (1) LT recipients without a persistent FEV_1_ decline (stable patients) and (2) LT recipients with a persistent decline of FEV_1_ < 80% of baseline. In the absence of confounding factors the diagnosis of CLAD was established and PFTs within 6 months were assessed to confirm the diagnosis.

During level-2 analysis, among CLAD cases, a decline of TLC to <90% of baseline was used to indicate RAS (8). When the number of available TLC values was insufficient for a confident diagnosis, a decline of FVC to <80% of baseline was used in combination with compatible imaging studies. For RAS cases, the FEV_1_/FVC ratio was calculated to identify a purely restrictive (≥0.70) or mixed ventilation defect (ratio <0.70). The final study population was re-assessed for an improvement of FEV1 of ≥10% after 3 months of macrolide therapy.

Patients were characterized as “Not stable/No definite CLAD” if: (1) they were not stable but did not have CLAD, i.e., deceased or retransplanted patients for causes other than CLAD (including patients who died within 3 months after LT) and (2) they had one or more confounding factors contributing to the decrease of PFTs. Cases with confounding factors were assessed in detail by the adjudication committee and whenever possible patient evolution was followed over time. These cases were characterized as “no definite CLAD” when the predefined diagnostic criteria of BOS or RAS were not met despite this detailed assessment. After this evaluation, three groups (stable, BOS, and RAS) were created and used as outcomes for the multivariate models.

### Collection of Predictors

Clinical variables were chosen for their relevance regarding CLAD (1, 12), and subsequent data harmonization targeted to overcome discrepancies between the cohorts and among different centers. With a focus on those objectives, the following variables were collected and served as predictors in the multivariate models: (a) recipients’ baseline characteristics, (b) donors’ baseline characteristics, (c) data on induction treatment and surgery, and (d) data on follow-up: stage 3 primary graft dysfunction (PGD), maintenance immunosuppression, number of treated acute cellular rejection episodes during year 1 (Y1 t-AR), number of treated infections during year 1 (Y1 t-infections), number of treated CMV infections during year 1 (Y1 t-CMV), and donor-specific antibodies (DSAs). Details for specific variables are provided below.

Human leukocyte antigen (HLA) mismatches concerned the sum of A1, A2, B1, B2, DR1, and DR2 mismatches between recipients and donors (maximum possible sum: 6). Baseline DQ mismatches were not included in this sum as they were not available for all patients since the creation of the two cohorts.

Regarding PGD, for each patient, the worse stage developing within the first 72 h post-operatively (26, 27) is captured in the databases. Charts were reviewed to confirm accurate characterization according to established criteria (PaO_2_/FiO_2_ ratio < 200, radiological infiltrates and/or ECMO). Although we were able to confirm severe PGD, milder PGD stages were difficult to establish unequivocally in all cases and for that reason they were not included in the analysis.

Y1 t-AR refers to the total number of treated events of grade A rejection or lymphocytic bronchiolitis (biopsy proven or clinically suspected). For harmonization reasons, we used this parameter instead of the cumulative A score [the latter depended on the frequency of conducted biopsies and the occurrence of Ax (inconclusive) results which were variable among centers].

Y1 t-infections refer to the total number of treated microbial, viral, and fungal infections (pulmonary and extra-pulmonary). Data collection especially for viral infections was not consistent between the databases and among centers, and for this reason, we did not use this variable. However, data on treated infections (including treated CMV infections) were recorded consistently and in detail.

Donor-specific antibody measurements were conducted at each study center. In order to alleviate methodological discrepancies, DSAs were considered positive when the result was validated as such by the corresponding immunology laboratory and no unique cut-off for mean fluorescence intensity (MFI) was used (Table S1 in Supplementary Material).

For some parameters, clinical practices, definitions, or data collection were very heterogeneous or inconsistent among centers, not allowing harmonization under one common definition. These parameters did not pass the quality control to be included in the analysis and were considered missing variables. Diagnosis of antibody-mediated rejection was not possible to establish with certainty for all patients since the creation of the cohorts, not only due to the evolving definition criteria but also due to the variability of clinical practices among centers and over time. The lack of an international standardization during data collection [International Society for Heart and Lung Transplantation (ISHLT) guideline published in 2016 (28)] led to subsequent discrepancies in the interpretation and management of positive results. Investigations and definitions of gastroesophageal reflux varied significantly among centers, depending on different local clinical practices and era of transplant. For that reason, it was not possible to harmonize this variable under one common definition.

### Data Quality Control and Harmonization

Data quality control strategies were implemented by three teams: (a) COLT coordinators, COLT datacenter (Informatique Données Base Centralisées—IDBC, St Luce/Loire), (b) STCS coordinators, STCS datacenter (Basel, Switzerland), and (c) SysCLAD coordinators, SysCLAD datacenter (Biomax, Germany).

Within each cohort, data were collected prospectively by the clinical research assistants according to the cohort-specific data dictionary and a predefined acquisition methodology. The corresponding datacenters conducted regular, automatic data quality validation assessments including but not restricted to: completeness, availability of the required data, data type check, data ranges based on realistic expected values, and rule-based inconsistency checks (e.g., diagnosis of “stable” is in conflict with an FEV1 < 80% of baseline). Database coordinators addressed datacenter originating queries to verify or complete data.

To identify inconsistencies in the definitions and in data completion between COLT and STCS, a harmonization working group (Christophe Pison, Laurent P. Nicod, Pierre J. Royer, Angela Koutsokera, and Andreas Fritz) defined which data were sufficiently harmonized, which needed refinement or completion before achieving harmonization, and which were not possible to harmonize (latter considered as missing variables). The conclusions and suggestions of this working team were presented at a regular basis to the SysCLAD consortium members who validated the final decisions. Details on the collected clinical parameters, quality control, and missing variables are reported in Tables S1–S3 in Supplementary Material.

### Statistical Analysis

Continuous variables were reported as median (IQR) and categorical as *n* (%). Mann–Whitney *U*-test was used for two-group comparisons, Kruskal–Wallis for three groups, and chi-square for categorical variables.

Univariate and multivariate logistic regression analyses (LRAs) were used to develop a prediction model for early-onset CLAD. Univariate and multivariate multinomial analyses were used to develop a prediction model for early-onset BOS or RAS. Independent explanatory variables were identified by backward–forward and forward–backward elimination techniques (29). The entry and removal criteria from the equation were a probability of likelihood ratio <0.05 and >0.10, respectively. The “center of transplantation” was coded as an indicator variable and, during multivariate analysis, systematically forced into the model as one of the explanatory variables to provide adjustment for center effect. For model performance and to test the overall fit of the models, we used the Hosmer–Lemeshow goodness-of-fit and the *R*^2^ (Cox–Snell and McFadden) tests. Results were reported as odds ratios (ORs, 95% CI).

Variables of the best performing multivariate LRA model were used to create a receiver operating characteristic (ROC) curve. The equation for the prediction of CLAD corresponding to this ROC curve was created for the studied population. Probabilities of stability, BOS, or RAS were calculated for all modalities of each significant independent variable in the multivariate multinomial analysis.

Cox proportional hazards analysis was used to identify risk factors for mortality in the studied population. Variables with *p*-value of 0.1 or less in the univariate analysis were included in the multivariate model. A backward/forward selection procedure with Akaike information criterion was used to identify risk factors for mortality, and survival curves were obtained from Kaplan–Meier estimates.

Statistical significance was defined as *p* < 0.05. All statistical analyses were performed using R 3.2.0 and SPSS 22 software versions.

## Results

### Study Population and Subgroups of Patients

As shown in Figure 1, from 1,263 LT included in COLT and STCS, 422 adult and pediatric recipients fulfilled the SysCLAD eligibility criteria. Following adjudication, a definite diagnosis was established for 230 subjects, transplanted in 12 different centers, and these patients were analyzed further. Patients’ characteristics are presented in Table 1.

**Table 1:** Characteristics of the studied population and comparisons between stable recipients and recipients diagnosed with BOS or RAS by 3 years post-LT.

		All patients (*n* = 230)	Stable recipients (*n* = 149)	BOS (*n* = 51)	RAS (*n* = 30)	*p*-Value
**Recipients’ characteristics**						
Age (years)		46.5 [29, 58]^a^	43 [30, 56]	47 [27, 57.5]	57 [38, 62]	0.0517
Gender	Male	118 (51.3)	78 (52)	23 (45.1)	17 (56.7)	0.55
BMI (kg/m^2^)		19.9 [17.9, 24.2]	19.6 [17.8, 23.7]	20.1 [17.9, 23.1]	23.6 [18.6, 26.8]	**0.0341**
Blood group	A	97 (42.4)	66 (44.3)	20 (40)	11 (36.7)	0.63
	AB	15 (6.6)	11 (7.4)	3 (6)	1 (3.3)	
	B	29 (12.7)	20 (13.4)	7 (14)	2 (6.7)	
	O	88 (38.4)	52 (34.9)	20 (40)	16 (53.3)	
Underlying diagnosis	COPD	61 (26.5)	37 (24.8)	14 (27.5)	10 (33.3)	**<0.001**
	CF	88 (38.3)	67 (45)	15 (29.4)	6 (20)	
	ILD/IPF	43 (18.7)	20 (13.4)	10 (19.6)	13 (43.3)	
	Others	38 (16.5)	25 (16.8)	12 (23.5)	1 (3.3)	
Smoking history	Yes	108 (47.8)	63 (43.2)	26 (52)	19 (63.3)	0.10
HLA mismatches		5 [4,5]	5 [4, 5]	5 [4, 5]	5 [4, 5.75]	0.81

**Donors’ characteristics**						
Age (years)		45 [32, 55]^b^	43 [31, 56]	47 [37.5, 54]	49 [37, 53]	0.61
Gender	Male	124 (53.9)	83 (55.7)	23 (45.1)	18 (60)	0.33
Blood group	A	95 (45.3)	64 (43)	21 (41.2)	10 (33.3)	0.89
	AB	10 (4.3)	6 (4)	3 (5.9)	1 (3.3)	
	B	29 (12.6)	19 (12.8)	7 (13.7)	3 (10)	
	O	96 (41.7)	60 (40.3)	20 (39.2)	16 (53.3)	
Smoking history	Yes	95 (42.8)	59 (40.7)	24 (50)	12 (41.4)	0.521

**Intervention**						
Type of intervention	Double lung	192 (83.5)	127 (85.2)	37 (72.5)	28 (93.3)	0.20
	Single lung	5 (2.2)	16 (10.7)	11 (21.6)	2 (6.7)	
	Lobar	4 (1.7)	2 (1.3)	2 (3.9)	0 (0)	
	Heart–lung	29 (12.6)	4 (2.7)	1 (2)	0 (0)	
Max cold ischemia time (min)		320 [275, 380]	330 [281, 380]	320 [247, 397]	304 [284, 371]	0.62
Induction treatment	Basiliximab	108 (47)	80 (53.7)	14 (27.5)	14 (46.7)	**0.0016**
	None	65 (28.3)	40 (26.8)	14 (27.5)	11 (36.7)	
	rATG	57 (24.8)	29 (19.5)	23 (45.1)	5 (16.7)	

**Follow-up**						
PGD stage 3	Severe	7 (3.1)	5 (3.4)	1 (2)	1 (3.3)	0.87
Immunosuppression	Cyclosporin	91 (39.6)	54 (36.2)	19 (37.3)	18 (60)	**0.0488**
	Tacrolimus	139 (60.4)	95 (63.8)	32 (62.7)	12 (40)	
Y1 t-AR		0 [0, 1]	0 [0, 1]	0[0, 1]	0[0, 1]	0.65
Y1 t-infections		1 [0, 2]	1 [0, 2]	1 [0, 2]	1 [0, 2]	0.99
Y1 t-CMV		0 [0, 0]	0 [0, 0]	0 [0, 1]	0 [0, 0]	0.37
DSA before LT	Yes	42 (20.3)	20 (14.9)	15 (33.3)	7 (25.0)	**0.024**
Y1 DSAs (I or II)	Yes	48 (21.3)	21 (14.3)	17 (35.4)	10 (33.3)	**0.002**
Y1 DSAs I	Yes	24 (10.6)	11 (7.5)	10 (20.4)	3 (10)	**0.039**
Y1 DSAs II	Yes	42 (18.7)	17 (11.6)	16 (33.3)	9 (30)	**0.001**

*Statistically significant results are highlighted in bold*.

*Continuous variables are presented as median [IQR] and categorical variables as *n* (%)*.

*^a^Recipient’s age range = 14–68 years*.

*^b^Donor’s age range = 10–78 years*.

*AR, acute cellular rejection episodes; BMI, body mass index; CF, cystic fibrosis; CMV, cytomegalovirus; COPD, chronic obstructive pulmonary disease; DSAs, donor-specific antibodies; HLA, human leukocyte antigen; ILD/IPF, interstitial lung disease/idiopathic pulmonary fibrosis; LT, lung transplantation; rATG, rabbit antithymocyte globulin; t, treated; PGD, primary graft dysfunction; Y1, year 1; BOS, bronchiolitis obliterans syndrome; RAS, restrictive allograft syndrome*.

The following groups were analyzed: (1) stable recipients (*n* = 149), CLAD (*n* = 81), and (2) CLAD patients were further subcategorized as BOS (51) or RAS (*n* = 30). Among the BOS patients, 17.6% were stage 1, 29.4% stage 2, and 53% stage 3. Concerning RAS, 37% (*n* = 11) had a FEV_1_/FVC ratio <0.70 indicating a mixed pattern (example case in Supplementary Material).

The final study population was assessed for an FEV_1_ improvement of at least 10% after 3 months of macrolide therapy (either azithromycin or clarithromycin). FEV_1_ of all CLAD patients remained below the 80% of baseline threshold, but six (7.4%) had a reversibility of at least 10% after 3 months of macrolides. Within the stable population, 74 (49.7%) received macrolides for at least 3 months (either for a non-sustained decline of FEV_1_ after an acute event, or as part of a treatment for non-tuberculous mycobacteria). Twenty-two patients (14.8% of the stable patients) had an improvement of at least 10% of FEV_1_ at 3 months of treatment. In all stable patients, FEV1 remained above the 80% of baseline threshold. In macrolide-responsive cases, the improvement of FEV_1_ could not be attributed to macrolides alone, since during the same period, most patients had concomitant treatments for a viral or bacterial infection or an acute cellular rejection.

Among the CLAD patients, 35 died (for 19, BOS or RAS was the main cause of death) and 8 were retransplanted (5 due to BOS and 3 due to RAS). The “Not stable/No definite CLAD” group included 147 patients who died or were retransplanted for causes other than CLAD and 45 patients with at least 1 confounding factor contributing to the decrease of FEV_1_ and/or TLC (detailed in Figure 1).

### Model Development and Model Regression Diagnostics

The parameters tested by backward–forward and forward–backward elimination techniques to identify independent explanatory variables were the following: recipient’s age, donor’s age, difference of recipient and donor’s age, underlying diagnosis, recipient’s smoking history before LT, recipient’s body mass index (BMI), stage 3 PGD, max cold ischemia time, number of HLA mismatches, induction treatment, maintenance immunosuppression (cyclosporine vs. tacrolimus), Y1 t-AR, Y1 t-infections, Y1 t-CMV infections, DSAs before LT, Y1 DSAs, Y1 DSAs class I, and Y1 DSAs class II.

For the analyzed parameters, only a small number of data was missing (Table S2 in Supplementary Material). Single imputation was used to handle missing data. For nominal variables, missing values were replaced by the variable mode (i.e., most frequent value), and for numerical variables, missing values were replaced by the median value. The only exception was “DSAs before LT” which had the most missing data (23 of 230). In this case, missing data were coded as unknown.

For induction treatment, we used “basiliximab” as the baseline and for “underlying diagnosis” we chose “CF.” For the former, a preliminary exploratory univariate analysis using “none” as the baseline did not provide statistically significant differences [basiliximab vs. none OR (95% CI) 0.882 (0.763, 1.020), *p* = 0.09 and rabbit antithymocyte globulin (rATG) vs. none 1.113 (0.941, 1.315), *p* = 0.214]. We considered that the statistically significant difference observed using basiliximab as the baseline was of clinical interest. In addition, basiliximab was the most frequently used induction agent within this study (Table S3 in Supplementary Material). The choice of CF as the baseline for the “underlying diagnosis” was done for the same reasons (statistical significant results in the preliminary analysis, most frequent diagnosis in the study population). We considered that this comparison would be of particular clinical interest.

With regard to model regression diagnostics: (a) the dependent variable was either binary (stable, BOS) or ordinal (stable, BOS, and RAS), (b) the dependent variables were coded appropriately, (c) the model fitted correctly after using a stepwise method, the Hosmer–Lemeshow goodness-of-fit and the *R*^2^ test, (d) the data originated from independent samples (no paired samples) and we used the stepwise approach to avoid multicollinearity, (e) linearity of independent variables and log odds was observed in the graphics diagnostics, and (f) we used a sample size of 230 cases to test 18 variables (i.e., more than 10 samples per independent variable).

### Prediction Model for Early-Onset CLAD

**Figure 2:**
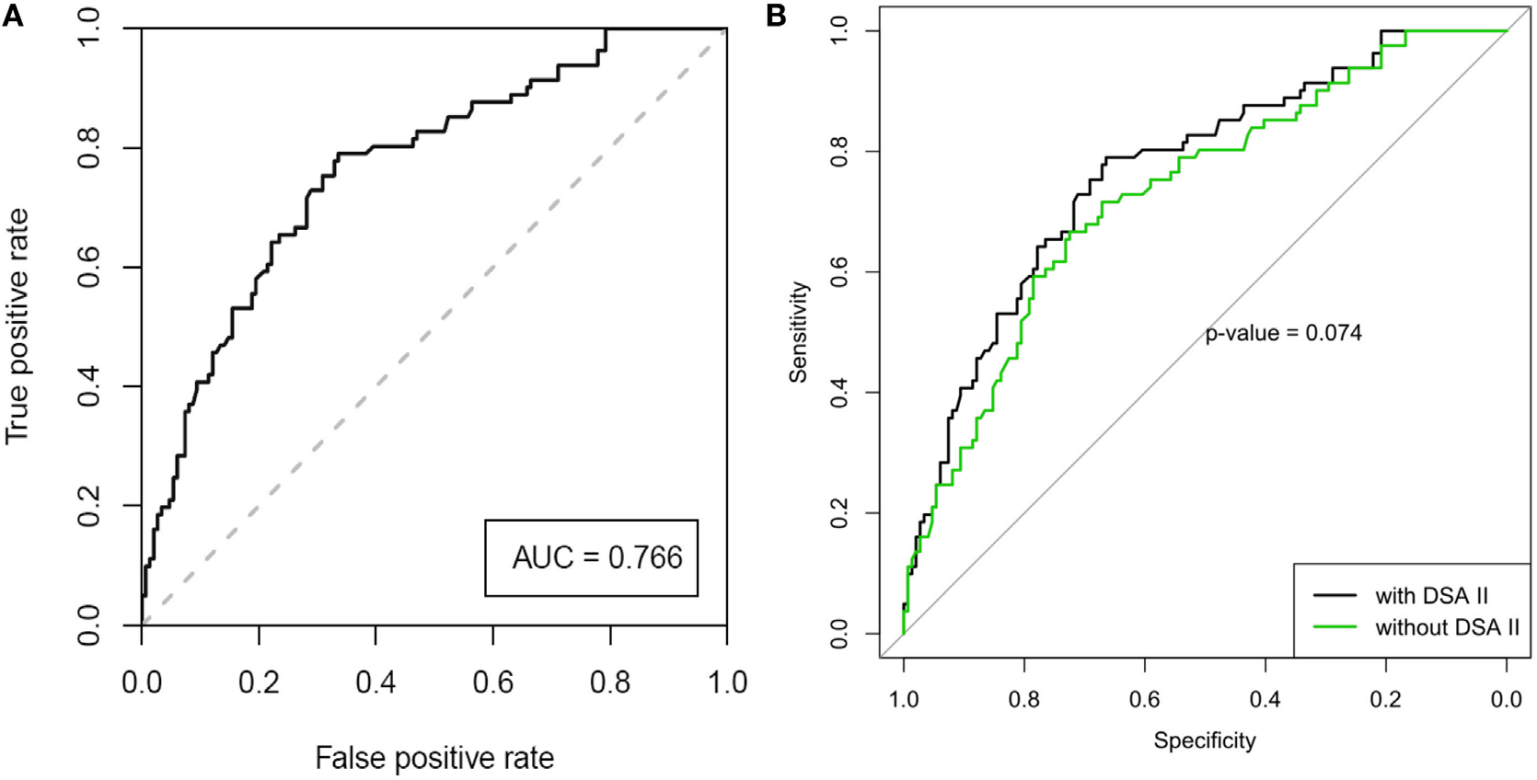
**(A)** Receiver operating characteristic (ROC) analysis of the best performing model for the prediction of early-onset chronic lung allograft dysfunction after adjusting for center effect [area under the curve (AUC) = 0.766, SE = 0.0325, 95% CI 0.703–0.830]. **(B)** ROC curve of the model, with and without the parameter of “Y1 class II DSA” (AUC 0.766 vs. 0.730, respectively, *p* = 0.074).

Table 2 shows the results of the univariate and multivariate LRA. The variables of the best performing multivariate LRA model for early-onset CLAD were used to create a multivariate prediction model for estimating probability of developing early-onset CLAD. The variables included recipient age, underlying diagnosis, induction treatment and presence of Y1 class II DSAs, and the prediction model generated a ROC curve (Figure 2A) with an area under the curve (AUC) of 0.766. The equation corresponding to this ROC curve, its limitations, and some clinical examples for the prediction of early-onset CLAD in the studied population are presented in Table S4 in Supplementary Material. The ROC curve of the same model but without using Y1 class II DSA provided an AUC = 0.730. The difference between the AUC of the complete model and the model without Y1 class II DSA did not reach statistical significance (*p* = 0.074, Figure 2B).

**Table 2:** Risk factors for the development of CLAD (multivariate analysis adjusted for center effect) by 3 years post-LT.

Variable		Univariate analysis		Multivariate analysis	
		OR (95% CI)	*p*-Value	OR (95% CI)	*p*-Value
Recipient age		1.002 (0.998, 1.006)	0.2288	0.971 (0.938, 1.006)	0.102
Donor age		1.002 (0.998, 1.006)	0.3172		
Difference of R/D age		1.000 (0.997, 1.004)	0.9041		
Recipient smoking	Yes	1.128 (0.996, 1.277)	0.0598		
Recipient BMI		1.012 (0.998, 1.025)	0.0924		
Underlying diagnosis	CF	Baseline		Baseline	
	COPD	1.167 (1.002, 1.361)	**0.049**	5.158 (1.444, 18.426)	**0.012**
	ILD/IPF	1.345 (1.133, 1.596)	**<0.001**	9.429 (2.291, 38.807)	**0.002**
	Others	1.109 (0.928, 1.326)	0.2573	2.435 (0.783, 7.569)	0.124
Sum of HLA mismatches		1.011 (0.948, 1.077)	0.7396		
Max cold ischemia time		1.000 (0.999, 1.001)	0.6245		
Induction treatment	Basiliximab	Baseline		Baseline	
	None	1.134 (0.981, 1.310)	0.0914	1.382 (0.451, 4.236)	0.572
	rATG	1.261 (1.084, 1.467)	**0.0029**	3.519 (0.946, 13.085)	0.060
PGD stage 3	Yes	0.928 (0.646, 1.333)	0.6853		
Immunosuppression	Cyclosporin	Baseline			
	Tacrolimus	0.914 (0.805, 1.037)	0.1635		
Y1 t-AR		1.003 (0.938, 1.072)	0.9392		
Y1 t-infections		1.003 (0.970, 1.036)	0.8784		
Y1 t-CMV		1.019 (0.899, 1.155)	0.764		
DSA before LT	Yes	1.240 (1.056, 1.455)	**0.0092**		
Y1 DSAs (I or II)	Yes	1.316 (1.135, 1.526)	**0.0004**		
Y1 DSAs I	Yes	1.240 (1.014, 1.515)	**0.0370**		
Y1 DSAs II	Yes	1.357 (1.162, 1.585)	**0.0001**	4.221 (1.784, 9.991)	**0.001**

*Statistically significant results are highlighted in bold*.

*Results are presented as OR (95% confidence intervals). For binary variables, the “No” group has been considered the baseline group*.

*AR, acute cellular rejection episodes; BMI, body mass index; CF, cystic fibrosis; CMV, cytomegalovirus; COPD, chronic obstructive pulmonary disease; DSAs, donor-specific antibodies; HLA, human leukocyte antigen; ILD/IPF, interstitial lung disease/idiopathic pulmonary fibrosis; LT, lung transplantation; t, treated; PGD, primary graft dysfunction; rATG, rabbit antithymocyte globulin; Y1, year 1; CLAD, chronic lung allograft dysfunction; OR, odds ratio*.

Of note, increasing recipient age had a weak protective effect over CLAD development (OR 0.971, *p* = 0.102 in the multivariate LRA, factor −0.03 in the ROC curve equation). A graphic representation of early-onset CLAD diagnosis according to recipient age showed a tendency for a U-shaped distribution. However, when the recipients’ age was tested for a quadratic (i.e., non-linear) effect, *p*-value was not statistically significant (Figure S1 in Supplementary Material).

### Prediction Models for Early-Onset BOS or RAS

**Figure 3:**
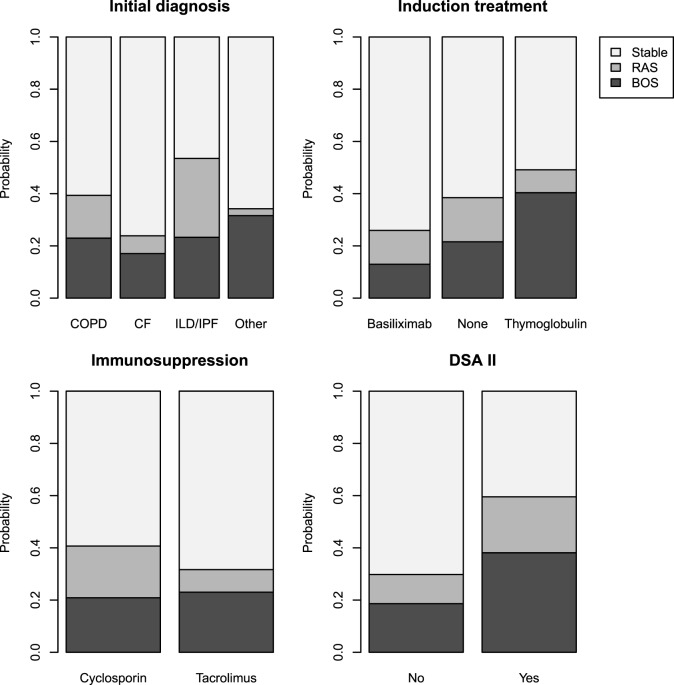
Probabilities of stability, bronchiolitis obliterans syndrome (BOS), and restrictive allograft syndrome (RAS) calculated for all modalities of each significant independent variable for the studied population (*n* = 230).

Tables 3 and 4 present the results of the univariate and multivariate multinomial analysis. The multivariate multinomial predictive model included the underlying diagnosis, maintenance immunosuppression, induction treatment, and Y1 class II DSAs. Within the model, Y1 class II DSAs were associated with BOS (OR 3.83) and RAS (OR 6.97). Compared to CF, interstitial lung disease/idiopathic pulmonary fibrosis (ILD/IPF) (OR 5.47) and chronic obstructive pulmonary disease (COPD) (OR 3.86) were associated with a higher risk for RAS. Induction treatment and maintenance immunosuppression were included in the best prediction model, although they were not statistically significant. Probabilities for BOS and RAS for each significant independent variable are shown in Figure 3.

**Table 3:** Risk factors for BOS and RAS by 3 years post-LT as compared to stable recipients (univariate multinomial analysis).

Variable		BOS		RAS	
		OR (95% CI)	*p*-value	OR (95% CI)	*p*-Value
Recipient age		1.000 (0.980, 1.021)	0.974	1.031 (1.003, 1.060)	**0.030**
Donor age		1.005 (0.985, 1.025)	0.623	1.015 (0.990, 1.040)	0.235
Difference of R/D age		0.996 (0.979, 1.014)	0.683	1.008 (0.987, 1.030)	0.433
Recipient smoking history	Yes	1.420 (0.750, 2.687)	0.282	2.358 (1.048, 5.303)	**0.038**
Recipient BMI		1.014 (0.944, 1.089)	0.704	1.107 (1.024, 1.197)	**0.011**
Underlying diagnosis	CF	Baseline		Baseline	
	COPD	1.690 (0.736, 3.882)	0.216	3.018 (1.016, 8.966)	**0.047**
	ILD/IPF	2.233 (0.870, 5.736)	0.095	7.258 (2.444, 21.559)	**<0.001**
	Other	2.144 (0.833, 5.207)	0.092	0.447 (0.051, 3.897)	0.466
Sum of HLA mismatches		0.973 (0.706, 1.342)	0.870	1.180 (0.771, 1.805)	0.446
Max cold ischemia time		1.000 (0.996, 1.003)	0.904	0.998 (0.994, 1.003)	0.472
Induction treatment	Basiliximab	Baseline		Baseline	
	None	2.000 (0.870, 4.598)	0.103	1.571 (0.654, 3.774)	0.312
	rATG	4.532 (2.060, 9.972)	**<0.001**	0.985 (0.326, 2.977)	0.979
PGD stage 3	Yes	0.576 (0.066, 5.050)	0.618	0.993 (0.112, 8.819)	0.995
Immunosuppression	Cyclosporin	Baseline		Baseline	
	Tacrolimus	0.957 (0.495, 1.850)	0.897	0.379 (0.170, 0.846)	**0.018**
Y1 t-AR		1.110 (0.802, 1.536)	0.530	0.826 (0.505, 1.352)	0.448
Y1 t-infections		0.962 (0.803, 1.153)	0.678	1.087 (0.904, 1.308)	0.375
Y1 t-CMV		1.303 (0.721, 2.355)	0.380	0.763 (0.304, 1.915)	0.565
DSA before LT	Yes	2.850 (1.305, 6.223)	**0.009**	1.900 (0.714, 5.055)	0.199
Y1 DSAs (I or II)	Yes	3.048 (1.450, 6.406)	**0.003**	3.048 (1.254, 7.409)	**0.014**
Y1 DSAs I	Yes	3.060 (1.214, 7.714)	**0.018**	1.394 (0.364, 5.332)	0.628
Y1 DSAs II	Yes	3.550 (1.631, 7.726)	**0.001**	3.328 (1.313, 8.434)	**0.011**

*Statistically significant results are highlighted in bold*.

*Results are presented as OR (95% confidence intervals). For binary variables, the “No” group has been considered the baseline group*.

*AR, acute cellular rejection episodes; BMI, body mass index; CF, cystic fibrosis; CMV, cytomegalovirus; COPD, chronic obstructive pulmonary disease; D, donor; DSAs, donor-specific antibodies; HLA, human leukocyte antigen; ILD/IPF, interstitial lung disease/idiopathic pulmonary fibrosis; LT, lung transplantation; R, recipient; t, treated; PGD, primary graft dysfunction; rATG, rabbit antithymocyte globulin; Y1, year 1; BOS, bronchiolitis obliterans syndrome; RAS, restrictive allograft syndrome; OR, odds ratio*.

**Table 4:** Risk factors for BOS and RAS by 3 years post-LT as compared to stable recipients (multivariate multinomial analysis).

Variable		BOS		RAS	
		OR (95% CI)	*p*-Value	OR (95% CI)	*p*-Value
Underlying diagnosis	CF	Baseline		Baseline	
	COPD	1.606 (0.559, 4.610)	0.379	3.857 (1.041, 14.289)	**0.043**
	ILD/IPF	2.436 (0.738, 8.036)	0.144	5.467 (1.482, 20.170)	**0.011**
	Other	2.589 (0.848, 7.905)	0.095	0.230 (0.022, 2.398)	0.219
Immunosuppression	Cyclosporin	Baseline		Baseline	
	Tacrolimus	3.179 (0.704, 14.357)	0.133	0.670 (0.080, 5.590)	0.711
Induction treatment	Basiliximab	Baseline		Baseline	
	None	0.541 (0.117, 2.505)	0.432	4.528 (0.888, 23.076)	0.069
	rATG	3.101 (0.681, 14.123)	0.144	2.393 (0.307, 18.674)	0.405
Y1 DSAs II	Yes	3.827 (1.459, 10.040)	**0.006**	6.965 (1.839, 26.376)	**0.004**

*Statistically significant results are highlighted in bold*.

*Results are presented as OR (95% confidence intervals). For binary variables, the “No” group has been considered the baseline group*.

*CF, cystic fibrosis; COPD, chronic obstructive pulmonary disease; DSAs, donor-specific antibodies; ILD/IPF, interstitial lung disease/idiopathic pulmonary fibrosis; rATG, rabbit antithymocyte globulin; t, treated; Y1, year 1; BOS, bronchiolitis obliterans syndrome; RAS, restrictive allograft syndrome; OR, odds ratio*.

### Survival Analysis

Risk factors for mortality within 3 years after LT are shown in Table S5 in Supplementary Material, and survival curves obtained from Kaplan–Meier estimates are displayed in Figure S2 in Supplementary Material. In the multivariate analysis, the recipient previous smoking status, a diagnosis of IPF/ILD as compared to a diagnosis of CF, the sum of HLA mismatches, and the presence of DSA during year 1 (either class I or II) were associated with a higher risk of mortality.

## Discussion

This study analyzed clinical data of two large European LT cohorts aiming to develop multivariate prediction models for early-onset BOS or RAS. The created models provide probabilities for a binary outcome (stable vs. CLAD), not taking into account the probability of being in the “not stable/not CLAD” group. Our main findings are: first, the multivariate prediction model for CLAD included recipient age, underlying diagnosis, type of induction treatment, and Y1 class II DSAs. A model, using baseline variables only (i.e., exclusion of Y1 class II DSAs), had a predictive capacity similar to the complete model. Second, the multivariate prediction models for BOS and RAS included underlying diagnosis, type of induction treatment, maintenance immunosuppression, and Y1 class II DSAs, but were not identical. Year-1 class II DSAs were associated with both BOS and RAS, whereas pre-LT diagnoses of ILD/IPF and COPD were associated with RAS.

Establishment of a definite diagnosis of BOS or RAS is often challenging. Despite the detailed evaluation by the adjudication committee, 10% (*n* = 45) of the assessed patients had confounding factors not allowing a definite diagnosis. For the differential diagnosis of BOS and RAS, TLC is considered the gold standard; however, in case of insufficient TLC values, FVC may be used (1, 8, 25). Imaging studies are not included in the current diagnostic algorithms, but may support or contradict the PFT-based diagnosis. This may be particularly helpful when FVC decrease is associated with air trapping (pseudo-restriction) (30). The isolated evaluation of the FEV_1_/FVC ratio in these cases may be misleading and not allow identification of mixed ventilation defects. In our study, 37% (*n* = 11) of RAS patients had an FEV_1_/FVC ratio <0.70 suggesting a mixed PFTs pattern.

Prognostic prediction models for LT outcomes may assist patient risk stratification and improve follow-up strategies. Some previously described predictive models identified donor-specific risk factors for BOS (age ≥60 years, high PaO_2_, smoking, pulmonary infection, and HLA mismatch) (16) or recipient-related acute events occurring during follow-up (acute cellular rejection, infections, and fungal pathogens) (18, 19) as risk factors for BOS. So far, no study described a prediction model specifically for early-onset BOS and RAS. In our study, although factors of the BOS and RAS models were the same, significant differences were observed within the models. Y1 class II DSAs were associated with RAS and BOS, but the OR were 6.97 and 3.83, respectively. Compared to CF, ILD/IPF was COPD were associated with RAS. In the univariate analysis, use of rATG (vs. basiliximab) was a statistically significant risk factor for BOS, whereas tacrolimus was protective for RAS. Although neither induction nor maintenance immunosuppression were statistically significant in the prediction model, they both participated in it.

To date, no definite association between BOS development and underlying diagnoses has been established, but a shorter time to BOS has been reported for emphysema patients as compared to CF (31). Interestingly, IPF was associated with worse pulmonary function after BOS onset (32) and patients undergoing retransplantation for RAS were less likely to have CF and more likely to have IPF (33). In our study, as compared to CF, ILD/IPF (OR 5.467) and COPD (OR 3.857) were associated with RAS. To our knowledge, the later finding regarding COPD has not been previously described. Further studies are needed to confirm the underlying mechanisms of this association, but it can be hypothesized that pathological remodeling processes, as, for example, TGF-β signaling pathways or circulating fibroblasts, associated with ILD/IPF (34, 35) or COPD (36–39), may persist or be activated in some patients after LT and thus contribute to the higher risk of RAS.

Concerning recipients’ age and CLAD, different cutoff points have been used with discordant results (40–43). Data from the ISHLT Registry showed that 5-year incidence of BOS was 35.9% in pediatric and 41% adult patients (44, 45). In our study, the pediatric population was too small (3.5%) to be analyzed separately. A graphic representation of early-onset CLAD diagnosis according to recipient age showed a tendency for a U-shaped distribution, but this was not statistically significant. In the final model, increasing recipient age had a weak protective effect for CLAD, suggesting that age-related factors, such as worse adherence in children and adolescents or functional changes in the immune system during growth, may influence alloreactivity (46, 47). However, recipients’ age was not a component of the best predictive model for BOS or RAS. Regarding donors’ age, it was not associated with BOS/RAS or mortality; and, although literature is inconclusive (31, 48, 49), the observed lack of association in our cohort study is reassuring in regard to current donor selection criteria.

Evidence is not conclusive concerning induction treatment and the risk for CLAD (42, 43, 50–53), but recent data indicate that induction with basiliximab (or alemtuzumab) may be protective against BOS (54). In our study, use of basiliximab, as compared to rATG was also protective against BOS. It may be assumed that center effect or patient selection bias (e.g., high-risk patients receiving rATG) could account for the observed results. However, this factor remained significant even after controlling for center effect and independently of other perioperative risk factors (pre-LT DSAs, HLA mismatches, or cold ischemia time). Based on our results, causality cannot be established, but differences in the mechanisms of action of these molecules may explain our findings. The characteristics of post-depletion T cells and the susceptibility of individual T-cell subsets may vary between these agents (55–59). These results need to be further investigated as they concern a potentially modifiable factor.

Growing evidence suggests that DSAs are a risk factor for BOS and BOS-related mortality (31, 60, 61). DSAs that develop early after LT and persistent DSAs have been associated with worse outcomes (31). In our study, detection of type II DSAs at least once during year-1 was a risk factor of CLAD, BOS, and RAS consistently. As previously mentioned, important discrepancies existed in DSA measurements among different centers, reflecting a common problem of multicenter studies. In order to alleviate these discrepancies, we used the interpretation of the corresponding specialized laboratory rather than MFI cutoff points. Ongoing data collection in our cohort, specifically focusing on this parameter, is expected to provide additional information on this factor.

In line with evidence from transplantation of other solid organs, recent studies indicate that total HLA mismatches are associated with an increased risk for CLAD (54, 62). Hayes et al. described an increased risk for BOS, with HLA-A mismatches being associated with a greater hazard (54), whereas Walton et al. demonstrated the importance of eplet mismatches as a risk factor for RAS (62). In our study, HLA mismatches were an independent risk factor of mortality in the multivariate model but not for BOS or RAS. The observed discrepancy in these results may be due to differences in the definition of HLA mismatches and notably the non-inclusion of baseline DQ mismatches in our study since they were not available for all patients since the creation of the two cohorts.

Concerning other parameters, such as BMI (54), infections (63), or stage 3 PGD (64) and their association with BOS or RAS, data are limited. Clear associations may be hampered by the use of preemptive therapy or the decreased number of severe PGD survivors studied longitudinally. Acute cellular rejections (42, 65) and lymphocytic bronchiolitis (14, 66, 67) have been associated with BOS, but different definitions have been used in the literature. Concerning maintenance immunosuppression, two prospective studies showed a higher risk of BOS for cyclosporine vs. tacrolimus (68, 69). In our study, none of these clinical parameters had statistically significant associations with CLAD, with the exception of BMI and tacrolimus. BMI was associated with RAS in the univariate analysis but not in the multivariate analysis. For tacrolimus, a protective effect over RAS was shown in the univariate analysis, and this variable participated in the multivariate prediction model. Finally, for harmonization purposes, Y1-treated acute cellular rejections and lymphocytic bronchiolitis were studied together and Y1 t-AR was not an independent risk factor for BOS or RAS.

Our study has limitations. First, the choice of a 2-year minimum follow-up resulted in the exclusion of a large patient population from both cohorts. Although this approach may be associated with a patient selection bias, it allowed an unequivocal diagnosis in a number of patients sufficient for the construction of the models, while avoiding a bias associated with diagnostic uncertainty. Second, a specific time-point analysis was chosen over a time-dependent design to facilitate the subsequent multilevel clinical and laboratory data integration of the SysCLAD project. Follow-up was limited at 3 years because the main focus was early-onset CLAD. Although for RAS outcome seems to be inferior independently of the time-point of onset after LT, early BOS development has been associated with worse outcomes compared to late-onset BOS. Collectively, early-onset CLAD is associated with increased morbidity and mortality making the identification of associated risk factors even more clinically relevant. Finally, although there was not a significant number of missing values during model construction, very heterogeneous variables were not possible to harmonize (missing variables). These discrepancies reflect daily clinical practice and need to be taken into account when designing and analyzing multicenter databases. Adjudication of study patients and rigorous data harmonization are two of the main strengths of this project which differentiate it from registry studies handling more heterogeneous and difficult to control data (70). These results will need validation in a separate group of patients for whom data collection is ongoing within both cohorts.

In conclusion, the initial clinical data analysis of the SysCLAD cohort identified clinical factors as potential predictors of early-onset CLAD within 3 years post-LT. Among these factors, the underlying diagnosis, induction treatment, and the presence of Y1 class II DSAs were consistently associated with the development of CLAD and its main phenotypes, BOS or RAS. Validation of these results in subsequent patient populations and integration in the ongoing genetic, biological, and microbiological analyses of the SysCLAD project may assist patient risk stratification allowing a better understanding of the mechanisms propagating CLAD.

## SysCLAD Consortium

### Cohort of Lung Transplantation—COLT

***Bordeaux*:** J. Jougon, J-F. Velly, H. Rozé, E. Blanchard, C. Dromer; ***Bruxelles*:** M. Antoine, M. Cappello, M. Ruiz, Y. Sokolow, F. Vanden Eynden, G. Van Nooten, L. Barvais, J. Berré, S. Brimioulle, D. De Backer, J. Créteur, E. Engelman, I. Huybrechts, B. Ickx, T. J. C. Preiser, T. Tuna, L. Van Obberghe, N. Vancutsem, J-L. Vincent, P. De Vuyst, I. Etienne, F. Féry, F. Jacobs, C. Knoop, J. L. Vachiéry, P. Van den Borne, I. Wellemans, G. Amand, L. Collignon, M. Giroux; ***Grenoble*:** E. Arnaud-Crozat, V. Bach, P-Y. Brichon, P. Chaffanjon, O. Chavanon, A. de Lambert, J-P. Fleury, S. Guigard, R. Hacini, K. Hireche, A. Pirvu, P. Porcu, P. Albaladejo, C. Allègre, A. Bataillard, D. Bedague, E. Briot, M. Casez-Brasseur, D. Colas, G. Dessertaine, M. Durand, G. Francony, A. Hebrard, M. R. Marino, B. Oummahan, D. Protar, D. Rehm, S. Robin, M. Rossi-Blancher, C. Augier, P. Bedouch, A. Boignard, H. Bouvaist, E. Brambilla, A. Briault, B. Camara, J. Claustre, S. Chanoine, M. Dubuc, S. Quétant, J. Maurizi, P. Pavèse, C. Pison, C. Saint-Raymond, N. Wion, C. Chérion; ***Lyon*:** R. Grima, O. Jegaden, J-M. Maury, F. Tronc, C. Flamens, S. Paulus, J-F. Mornex, F. Philit, A. Senechal, -C. Glérant, S. Turquier, D. Gamondes, L. Chalabresse, F. Thivolet-Bejui, C Barnel, C. Dubois, A. Tiberghien; ***Paris, Hôpital Européen Georges Pompidou*:** F. Le Pimpec-Barthes, A. Bel, P. Mordant, P. Achouh, V. Boussaud, R. Guillemain, D. Méléard, M. O. Bricourt, B. Cholley, V. Pezella; ***Marseille*:** G. Brioude, X. B. D’Journo, C. Doddoli, P. Thomas, D. Trousse, S. Dizier, M. Leone, L. Papazian, F. Bregeon, A. Basire, B. Coltey, N. Dufeu, H. Dutau, S. Garcia, J. Y. Gaubert, C. Gomez, S. Laroumagne, A. Nieves, L. C. Picard, M. Reynaud-Gaubert, V. Secq, G. Mouton; ***Nantes*:** O. Baron, C. Brossaud, E. Durand, M. Durand, P. Lacoste, C. Perigaud, J. C. Roussel, I. Danner, A Haloun A. Magnan, A Tissot, T. Lepoivre, M. Treilhaud, K. Botturi-Cavaillès, S. Brouard, R. Danger, J. Loy, M. Morisset, M. Pain, S. Pares, D. Reboulleau, P.-J. Royer; ***Le Plessis Robinson, Hôpital Marie Lannelongue*:** Ph. Dartevelle, D. Fabre, E. Fadel, O. Mercier, S. Mussot, F. Stephan, P. Viard, J. Cerrina, P. Dorfmuller, S. Feuillet, M. Ghigna, Ph. Hervén F. Le Roy Ladurie, J. Le Pavec, V. Thomas de Montpreville, L. Lamrani; ***Paris, Hôpital Bichat*:** Y. Castier, P. Mordant, P. Cerceau, P. Augustin, S. Jean-Baptiste, S. Boudinet, P. Montravers, O. Brugière, G. Dauriat, G. Jébrak, H. Mal, A. Marceau, A-C. Métivier, G. Thabut, E. Lhuillier, C. Dupin, V. Bunel; ***Strasbourg*:** P. Falcoz, G. Massard, N. Santelmo, G. Ajob, O. Collange O. Helms, J. Hentz, A. Roche, B. Bakouboula, T. Degot, A. Dory, S. Hirschi, S. Ohlmann-Caillard, L. Kessler, R. Kessler, A. Schuller, B. Renaud-Picard, K. Bennedif, S. Vargas; ***Suresnes*:** P. Bonnette, A. Chapelier, P. Puyo, E. Sage, J. Bresson, V. Caille, C. Cerf, J. Devaquet, V. Dumans-Nizard, M. L. Felten, M. Fischler, A. G. Si Larbi, M. Leguen, L. Ley, N. Liu, G. Trebbia, S. De Miranda, B. Douvry, F. Gonin, D. Grenet, A. M. Hamid, H. Neveu, F. Parquin, C. Picard, A. Roux, M. Stern, F. Bouillioud, P. Cahen, M. Colombat, C. Dautricourt, M. Delahousse, B. D’Urso, J. Gravisse, A. Guth, S. Hillaire, P. Honderlick, M. Lequintrec, E. Longchampt, F. Mellot, A. Scherrer, L. Temagoult, L. Tricot, M. Vasse, C. Veyrie, L. Zemoura; ***Toulouse*:** J. Berjaud, L. Brouchet, M. Dahan, F. O. Mathe, H. Benahoua, M. DaCosta, I. Serres, V. Merlet-Dupuy, M. Grigoli, A. Didier, M. Murris, L. Crognier, O. Fourcade.

### Swiss Lung Transplant Centers

***Lausanne-Geneva*:** T. Krueger, H. B. Ris, M. Gonzalez, J.-D. Aubert, L. P. Nicod, B. J. Marsland, C. Berutto, T. Rochat, P. Soccal, Ph. Jolliet, A. Koutsokera, C. Marcucci, O. Manuel, E. Bernasconi, M. Chollet, F. Gronchi, C. Courbon; ***Zurich*:** S. Hillinger, I. Inci, P. Kestenholz, W. Weder; R. Schuepbach, M. Zalunardo, C. Benden, U. Buergi, L. C. Huber, B. Isenring, M. M. Schuurmans, A. Gaspert, D. Holzmann, N. Müller, T. Rechsteiner, C. Schmid, B. Vrugt.

We would like to thank the following collaborators of the Swiss Lung Transplantation Centers for their contribution in data collection and/or project coordination (alphabetical order): E. Catana, C. Cowaloosur-Noirat, M. F. Derkenne, JL Dreifuss, P. Grendelmeier, J. Hartwig, N. Lourenco, M. Magno, H. Muller-McKenna, E. Perret, and K. Zangger.

### Swiss Transplant Cohort Study—STCS

The members of the Swiss Transplant Cohort Study are: Rita Achermann, Patrizia Amico, John-David Aubert, Philippe Baumann, Guido Beldi, Christian Benden, Christoph Berger, Isabelle Binet, Pierre-Yves Bochud, Elsa Boely, Heiner Bucher, Leo Bühler, Thierry Carell, Emmanuelle Catana, Yves Chalandon, Sabina de Geest, Olivier de Rougemont, Michael Dickenmann, Michel Duchosal, Laure Elkrief, Thomas Fehr, Sylvie Ferrari-Lacraz, Christian Garzoni, Paola Gasche Soccal, Christophe Gaudet, Emiliano Giostra, Déla Golshayan, Karine Hadaya, Jörg Halter, Dominik Heim, Christoph Hess, Sven Hillinger, Hans H. Hirsch, Günther Hofbauer, Uyen Huynh-Do, Franz Immer, Richard Klaghofer, Michael Koller (Head of the data center), Bettina Laesser, Roger Lehmann, Christian Lovis, Oriol Manuel, Hans-Peter Marti, Pierre Yves Martin, Luca Martinolli, Pascal Meylan, (Head, Biological samples management group), Paul Mohacsi, Philippe Morel, Ulrike Mueller, Nicolas J. Mueller (Chairman Scientific Committee), Helen Mueller-McKenna (Head of local data management), Antonia Müller, Thomas Müller, Beat Müllhaupt, David Nadal, Manuel Pascual (Executive office), Jakob Passweg, Juliane Rick, Eddy Roosnek, Anne Rosselet, Silvia Rothlin, Frank Ruschitzka, Urs Schanz, Stefan Schaub, Aurelia Schnyder, Christian Seiler, Susanne Stampf, Jürg Steiger (Head, Executive Office), Guido Stirnimann, Christian Toso, Christian Van Delden (Executive office), Jean-Pierre Venetz, Jean Villard, Madeleine Wick (STCS coordinator), Markus Wilhelm, and Patrick Yerly.

### SME and Platforms

***Biomax(Munich*, *Germany)*:** A. Fritz, D. Maier; ***Finovatis (Lyon, France)*:** K. Desplanche, D. Koubi; ***GATC*** (***Germany)*:** F. Ernst, T. Paprotka, M. Schmitt, B. Wahl; ***Novasdicovery (Lyon, France)*:** J.-P. Boissel, G. Olivera-Botello; ***Prométhée Proteomics Platform (Grenoble, France)*:** C. Trocmé, B. Toussaint, S. Bourgoin-Voillard, M. Séve; ***Inserm U823, Université Joseph Fourier (Grenoble, France)*:** M. Benmerad, V. Siroux, R. Slama; ***European Institute for Systems Biology & Medicine (Lyon, France)*:** C. Auffray, D. Charron, J. Pellet, C. Pison.

## Ethics Statement

This study was carried out in accordance with the recommendations of national and local ethics committees of the participating cohorts with written informed consent from all subjects. All subjects gave written informed consent in accordance with the Declaration of Helsinki. The protocol was approved by the national and local ethics committees of the participating hospitals.

## Author Contributions

AK, PR, AF, AT, and KB performed data collection and harmonization. CB, JDA, AR, MR-G, AM, CP, and LN participated in the adjudication committee. JPA and AK performed the statistical analysis. AK drafted the manuscript. CB, JDA, AR, MR-G, RK, CD, SM, HM, J-FM, RG, CK, MD, PS, JC, ES, CG, AM, CP, and LN included the patients and revised the manuscript for important intellectual content.

## Conflict of Interest Statement

CP received fees for symposia and financial support to attend medical meetings from Astellas, Novartis, Sanofi-Genzyme, and Therakos. AR has served as a consultant for Novartis France concerning CMV in solid organ transplantation. J-FM acknowledges consultancy, research grant and support for meeting attendance from LFB Biomedicament, CSL Behring, Actelion, Pfizer, Pioproject, and Bayer. The authors have declared that no competing interests exist.

## Abbreviations

AIC, akaike information criterion; AUC, area under the curve; BMI, body mass index; BOS, bronchiolitis obliterans syndrome; CLAD, chronic lung allograft dysfunction; DSAs, donor-specific antibodies; FEV_1_, forced expiratory volume in one second; FVC, forced vital capacity; HLA, human leukocyte antigen; ISHLT, International Society for Heart and Lung Transplantation; ILD/IPF, interstitial lung disease, idiopathic pulmonary fibrosis; LRA, logistic regression analysis; LT, lung transplantation; PGD, primary graft dysfunction; OR, odds ratio; RAS, restrictive allograft syndrome; rATG, rabbit antithymocyte globulin; ROC, receiver operating characteristic; t-AR, treated acute rejections; t-CMV, treated CMV infections; t-infections, treated infections; TLC, total lung capacity.
