# Supplementary material for: Development of a Multivariate Prediction Model for Early-Onset Bronchiolitis Obliterans Syndrome and Restrictive Allograft Syndrome in Lung Transplantation
Source: Front Med (Lausanne). 2017 Jul 17;4:109. doi: 10.3389/fmed.2017.00109 (PMC5511826; doi:10.3389/fmed.2017.00109)
Supplement: Supplementary file 1 [file Table_1.DOCX]

**Table S1:** DSA measurement practices in the participating centers

| **Center** | **Technique** | **Mixed Ag screening or Single Ag** | **Assessment time points after LT** | **Positivity cut-off (MFI)** | **Change in methodology/practice according to the era** |
| --- | --- | --- | --- | --- | --- |
| **Bordeaux** | One Lambda |  | D10, D40, M3, M6, M12, annually. Frequency of measurements increased if DSA positivity | 500 |  |
| **Grenoble** | Immucor |  | M1, M3, M6, M12, annually. Frequency of measurements increased if DSA positivity | Any detected DSAs* |  |
| **Lausanne-Geneva** | One Lambda | Both | M1, M6, M12, annually. Frequency of measurements increased if DSA positivity | Until 2012: 2000  After 2012: 1000 | **2012:** Change in MFI cut-off  **2011:** measurement of donor DQ |
| **Le Plessis Robinson** | One Lambda | Single Ag | D0, D7, M1, M3, M6, M12 and when clinically indicated | 500 |  |
| **Lyon** | Immucor | Single Ag if positive screening | Each 2 months and after each immunizing event | 1000 | No |
| **Marseille** | One Lambda | Single Ag if positive screening | Before LT, D1, D8, D15, D21, D30, M3, M6, M9, M12, M15, M18, M21, M24, M30, M36, M48, M60 and when clinically indicated | 500 | No |
| **Nantes** | One Lambda | Both | Every week during M1, every month during 6M, then annually. Frequency of measurements increased if DSA positivity. | 2000 |  |
| **Paris-Bichat** | One Lambda | Single Ag | Before LT, D1, D15, M1, M3, M6, at each visit (every 3-6M). Frequency of measurements increased if DSA positivity | 500 | Gradually increased the frequency of DSA measurement |
| **Paris-HEGP** | One Lambda | Single Ag | D7/D15/D21/D30 M2, M3, M4 M5, M6 M9, M12 then each 6 month and when clinically indicated | 500 | Less screening and more Single Ag |
| **Strasbourg** | One Lambda | Singel Ag | D0, D10 and/or 21, M3, M12, then once a year and when clinically indicated | 1000 |  |
| **Suresnes** | One Lambda | Single Ag | Before LT, D1, D7, D21, M1, M2, M3, M4, M5, M6, M9, M12 then each 6 months and when clinically indicated | 500 | **2008-2012:** Mixed Ag screening, if positive test with single Ag  **2012-2015**le Ag only post LTx |
| **Zurich** | One Lambda | Both | M1, M3, M6, M12, annually. Frequency of measurements increased if DSA positivity | 500 | **2008-2012:** Mixed Ag screening, if positive test with single Ag  **2012-2015:** Single Ag at listing, during list waiting single Ag annually and mixed Ag screening every 6M, single Ag after sensitizing events |

* for immucor LSA, results are expressed as MFI normalized by background (negative control bead)

**Abbreviations**: Ag=antigen, D=day, DSAs=donor specific antibodies, LT=lung transplantation, MFI=mean fluorescent intensity, M=month, Y= year
