# Supplementary material for: Development of a Multivariate Prediction Model for Early-Onset Bronchiolitis Obliterans Syndrome and Restrictive Allograft Syndrome in Lung Transplantation
Source: Front Med (Lausanne). 2017 Jul 17;4:109. doi: 10.3389/fmed.2017.00109 (PMC5511826; doi:10.3389/fmed.2017.00109)
Supplement: Supplementary file 2 [file Table_2.DOCX]

**Table S2:** Missing data per patient and per variable for the studied population

| **Total number of missing variables** | 0 | 1 | 2 | 3 | 4 |
| --- | --- | --- | --- | --- | --- |
| **Number of patients** | 177 | 41 | 5 | 3 | 1 |

| **Variables** | **Number of missing data** |
| --- | --- |
| **Recipients’ characteristics** |  |
| Age | 0 |
| Gender | 0 |
| BMI | 0 |
| Blood group | 1 |
| Underlying diagnosis | 0 |
| Smoking history | 4 |
| HLA mismatches | 7 |
| **Donors’ characteristics** |  |
| Age | 0 |
| Gender | 0 |
| Blood group | 0 |
| Smoking history | 8 |
| **Intervention** |  |
| Type of intervention | 0 |
| Max cold ischemia time | 5 |
| Induction treatment | 0 |
| **Follow-up** |  |
| PGD stage 3 | 4 |
| Maintenance immunosuppression | 0 |
| Y1 t-AR | 0 |
| Y1 t-infections | 0 |
| Y1 t-CMV | 3 |
| DSAs before LT | 23 |
| Y1 DSAs I | 4 |
| Y1 DSAs II | 5 |
| **Outcome (stable, BOS, RAS)** | 0 |

**Abbreviations:** AR=acute cellular rejection episodes, BMI=body mass index, CMV=cytomegalovirus, DSAs=donor specific antibodies, HLA=human leukocyte antigen, LT= lung transplantation, t=treated, PGD=primary graft dysfunction, Y1=year 1
