# Supplementary material for: Development of a Multivariate Prediction Model for Early-Onset Bronchiolitis Obliterans Syndrome and Restrictive Allograft Syndrome in Lung Transplantation
Source: Front Med (Lausanne). 2017 Jul 17;4:109. doi: 10.3389/fmed.2017.00109 (PMC5511826; doi:10.3389/fmed.2017.00109)
Supplement: Supplementary file 3 [file Table_3.DOCX]

**Table S3:** Distribution of treatment modalities and patient phenotypes across centers

| **Center** | **Induction treatment** | **Immunosuppression** | **Stable (n=149)** | **BOS (n=51)** | **RAS/Mixed**  **(n=30)** |
| --- | --- | --- | --- | --- | --- |
| **Bordeaux** | Basiliximab n=0  rATG n=9  None n=9 | Tacrolimus n=1  Cyclosporin n=17 | 10 | 8 | 0 |
| **Grenoble** | Basiliximab n=6  rATG n=3  None n=0 | Tacrolimus n=9  Cyclosporin n=0 | 8 | 0 | 1 |
| **Lausanne-Geneva** | Basiliximab n=27  rATG n=0  None n=0 | Tacrolimus n=27  Cyclosporin n=0 | 21 | 3 | 2/1 |
| **Le Plessis Robinson** | Basiliximab n=0  rATG n=0  None n=11 | Tacrolimus n=1  Cyclosporin n=10 | 6 | 3 | 1/1 |
| **Lyon** | Basiliximab n=10  rATG n=0  None n=0 | Tacrolimus n=10  Cyclosporin n=0 | 10 | 0 | 0 |
| **Marseille** | Basiliximab n=1  rATG n=27  None n=3 | Tacrolimus n=31  Cyclosporin n=0 | 17 | 10 | 3/1 |
| **Nantes** | Basiliximab n=4  rATG n=9  None n=0 | Tacrolimus n=3  Cyclosporin n=10 | 7 | 5 | 1 |
| **Paris-Bichat** | Basiliximab n=0  rATG n=0  None n=10 | Tacrolimus n=8  Cyclosporin n=2 | 4 | 5 | 0/1 |
| **Paris-HEGP** | Basiliximab n=2  rATG n=2  None n=1 | Tacrolimus n=5  Cyclosporin n=0 | 2 | 3 | 0 |
| **Strasbourg** | Basiliximab n=15  rATG n=0  None n=4 | Tacrolimus n=19  Cyclosporin n=0 | 14 | 4 | 0/1 |
| **Suresnes** | Basiliximab n=4  rATG n=6  None n=19 | Tacrolimus n=24  Cyclosporin n=5 | 21 | 5 | 3 |
| **Zurich** | Basiliximab n=39  rATG n=1  None n=8 | Tacrolimus n=1  Cyclosporin n=47 | 29 | 5 | 8/6 |

Comment: In accordance to the current definition of BOS (BOS diagnosis established in the absence of a restrictive defect) cases with a mixed PFTs pattern were analyzed as RAS. The high number of definite RAS cases identified in Zurich was attributed to the large number of available TLC measurements in this center which allowed diagnostic certainty in many cases (fewer unclassified patients). rATG= rabbit antithymocyte globulin
