# Supplementary material for: Development of a Multivariate Prediction Model for Early-Onset Bronchiolitis Obliterans Syndrome and Restrictive Allograft Syndrome in Lung Transplantation
Source: Front Med (Lausanne). 2017 Jul 17;4:109. doi: 10.3389/fmed.2017.00109 (PMC5511826; doi:10.3389/fmed.2017.00109)
Supplement: Supplementary file 4 [file Table_4.DOCX]

**Table S4:** Equation calculating the probability of early onset CLAD (BOS or RAS) in the studied population, irrespective of the lung transplantation center. The recipients’ age was tested for a quadratic (i.e. non linear) effect but this was not statistically significant. This equation calculates probabilities for a binary outcome (stable vs CLAD) without taking into account the *unstable/no definite CLAD* patients. Patients who develop CLAD within the first year after LT were not excluded from these models. A simplified model, using baseline variables only (i.e. exclusion of Y1 class II DSAs), had a predictive capacity similar to the complete model.

| **Equation:**  **Probability of early onset CLAD = Exp (beta) / [1+Exp(beta)]**  **Beta =** - 0.933  - 0.030 x Recipient age at transplantation  + 0 (for CF) + 1.477 (for COPD) + 2.498 (for ILD/IPF) + 0.960 (for Other)  + 0 (for Basiliximab) + 0.282 (for No induction treatment) + 1.008 (for rATG)  + 1.189 (for DSAs class II during year-1) |
| --- |
| **Example 1:** 55-year old COPD patient who received rATG and developed DSAs class II during year-1 of follow-up  Beta = 1.091  Probability = 0.749 or 74.9% to be diagnosed with CLAD within 3 years of follow-up |
| **Example 2:** 20-year old CF patient who received basiliximab and did not develop DSAs class II during year-1 of follow-up  Beta = -1.533  Probability = 0.178 or 17.8% to be diagnosed with CLAD within 3 years of follow-up |

**Abbreviations :** DSA=donor specific antibodies, ILD/IPF=interstitial lung disease/idiopathic pulmonary fibrosis, rATG= rabbit antithymocyte globulin
