# Supplementary material for: Development of a Multivariate Prediction Model for Early-Onset Bronchiolitis Obliterans Syndrome and Restrictive Allograft Syndrome in Lung Transplantation
Source: Front Med (Lausanne). 2017 Jul 17;4:109. doi: 10.3389/fmed.2017.00109 (PMC5511826; doi:10.3389/fmed.2017.00109)
Supplement: Supplementary file 5 [file Table_5.DOCX]

**Table S5: Risk factors for mortality within 3 years after LT for the studied population**

|  |  | **Univariate analysis** | | **Multivariate analysis** | |
| --- | --- | --- | --- | --- | --- |
| **Variable** |  | **HR (95% CI)** | **p-value** | **HR (95% CI)** | **p-value** |
| Recipient age |  | 1.024 (1.000, 1.049) | **0.047** |  |  |
| Donor age |  | 1.003 (0.982, 1.024) | 0.788 |  |  |
| Difference of R/D age |  | 1.013 (0.995, 1.031) | 0.154 |  |  |
| Recipient smoking | Yes | 3.167 (1.501, 6.680) | **0.002** | 2.687 (1.179, 6.126) | **0.019** |
| Recipient BMI |  | 1.054 (0.986, 1.126) | 0.122 |  |  |
| Underlying diagnosis | CF | Baseline |  | Baseline |  |
|  | COPD | 1.694 (0.666, 4.308) | 0.268 | 0.884 (0.302, 2.592) | 0.822 |
|  | ILD/IPF | 3.446 (1.441, 8.238) | **0.005** | 3.109 (1.162, 8.320) | **0.024** |
|  | Other | 1.420 (0.476, 4.247) | 0.531 | 1.047 (0.335, 3.277) | 0.937 |
| Sum of HLA mismatches |  | 1.401 (0.954, 2.056) | 0.085 | 1.621 (1.077, 2.439) | **0.021** |
| Max cold ischemia time |  | 1.002 (0.998, 1.006) | 0.298 |  |  |
| Induction treatment | Basiliximab | Baseline |  |  |  |
|  | None | 0.832 (0.397, 1.746) | 0.627 |  |  |
|  | rATG | 0.407 (1.149, 1.111) | 0.079 |  |  |
| PGD stage 3 | Yes | 0.448 (0.026, 7.652) | 0.580 |  |  |
| Immunosuppression | Cyclosporin | Baseline |  |  |  |
|  | Tacrolimus | 0.696 (0.359, 1.353) | 0.285 |  |  |
| Y1 t-AR |  | 0.817 (0.532, 1.255) | 0.355 |  |  |
| Y1 t-infections |  | 1.116 (0.959, 1.299) | 0.155 |  |  |
| Y1 t-CMV |  | 0.654 (0.297, 1.449) | 0.295 |  |  |
| DSAs before LT | Yes | 0.942 (0.383, 2.316) | 0.897 |  |  |
| Y1 DSAs (I or II) | Yes | 2.900 91.447, 5.815) | **0.003** | 2.952 (1.430, 6.094) | **0.003** |
| Y1 DSAs I | Yes | 1.858 (0.764, 4.518) | 0.172 |  |  |
| Y1 DSAs II | Yes | 2.861 (1.414, 5.789) | **0.003** |  |  |

Results are presented as hazard-ratio (95% confidence intervals). For binary variables, the ‘No’ group has been considered the baseline group.

**Abbreviations**: AR= acute cellular rejection episodes, BMI=body mass index, CF= cystic fibrosis, CMV=cytomegalovirus, COPD=chronic obstructive pulmonary disease, DSAs=donor specific antibodies, HLA=human leukocyte antigen, ILD/IPF=interstitial lung disease / idiopathic pulmonary fibrosis, LT= lung transplantation, t=treated, PGD=primary graft dysfunction, Y1= year 1
