# Supplementary material for: Development of a Multivariate Prediction Model for Early-Onset Bronchiolitis Obliterans Syndrome and Restrictive Allograft Syndrome in Lung Transplantation
Source: Front Med (Lausanne). 2017 Jul 17;4:109. doi: 10.3389/fmed.2017.00109 (PMC5511826; doi:10.3389/fmed.2017.00109)

Example case: PFTs and imaging studies in a case of RAS with mixed restrictive and obstructive pattern

- **Evolution of FEV1, FVC and TLC during follow-up**

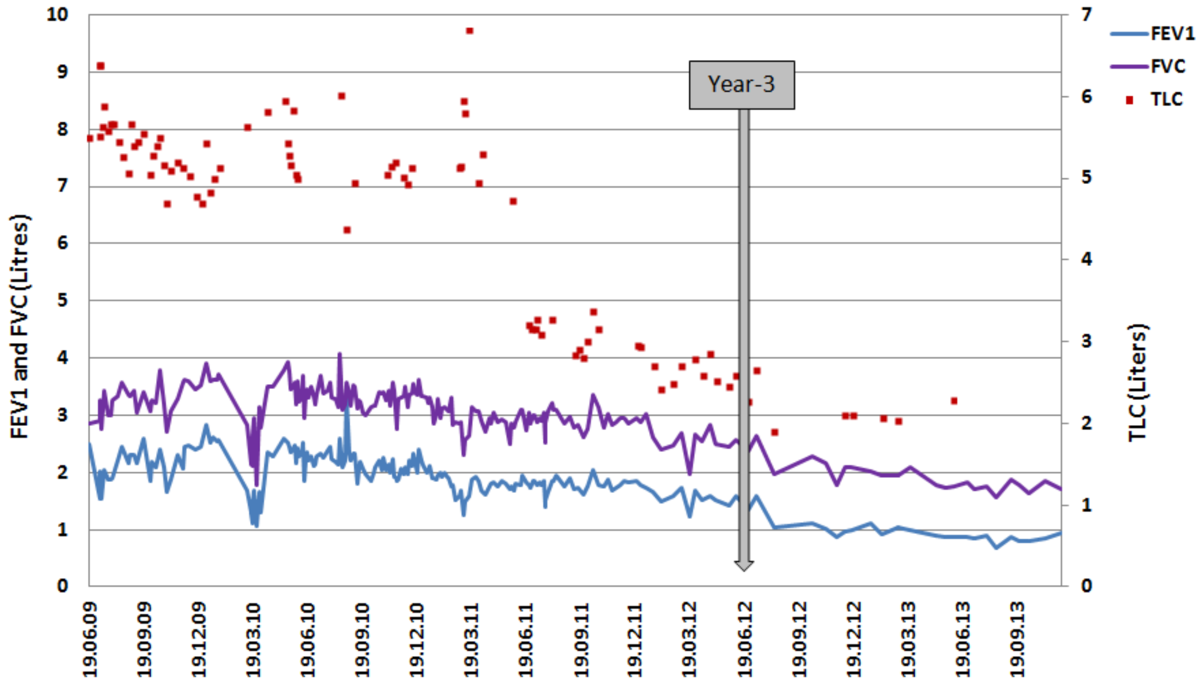

- **Values at the time-point of year 3:**
  FEV_1 % of baseline = 53%
  FVC % of baseline = 61%
  TLC % of baseline = 25%
  FEV_1/FVC = 0.55

- **Computed tomography of the thorax in the same patient showing apical fibrosis and an interstitial pattern compatible with RAS**

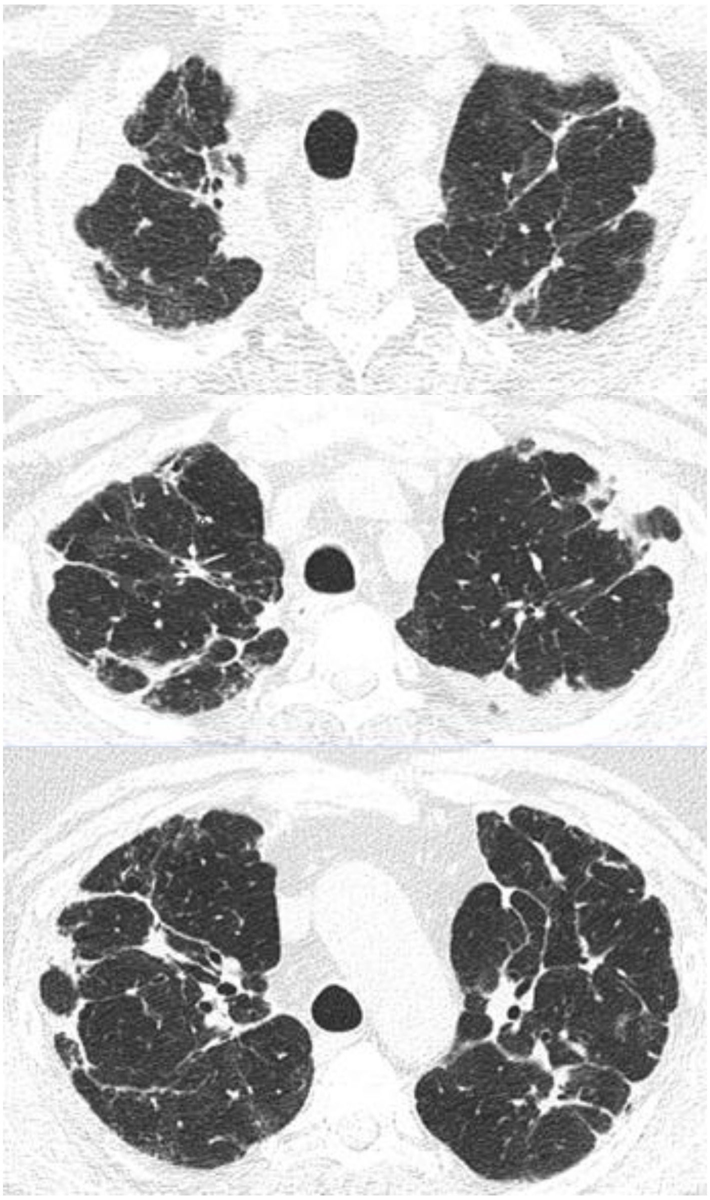

Supplement: Supplementary file 6 [file Presentation_1.PDF]
