# Supplementary material for: Development of a Multivariate Prediction Model for Early-Onset Bronchiolitis Obliterans Syndrome and Restrictive Allograft Syndrome in Lung Transplantation
Source: Front Med (Lausanne). 2017 Jul 17;4:109. doi: 10.3389/fmed.2017.00109 (PMC5511826; doi:10.3389/fmed.2017.00109)
Supplement: Supplementary file 7 [file Image_1.PDF]

Figure S1: a) Proportion of early onset CLAD and stability according to recipient age subgroups, b) Proportion of early onset BOS, RAS and stability according to recipient age subgroups. Although there was a tendency of a non-linear effect of the recipients' age in regard to CLAD, testing for a quadratic association (i.e. no linear effect) did not reach statistical significance.

a)

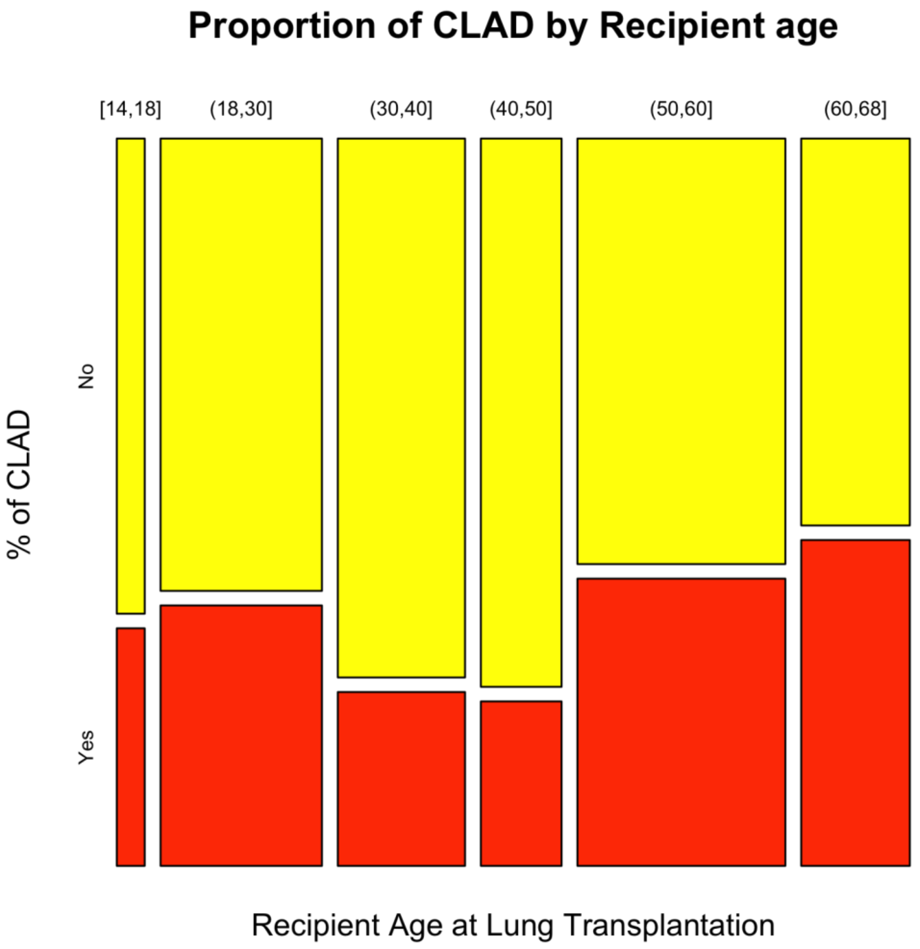

b)

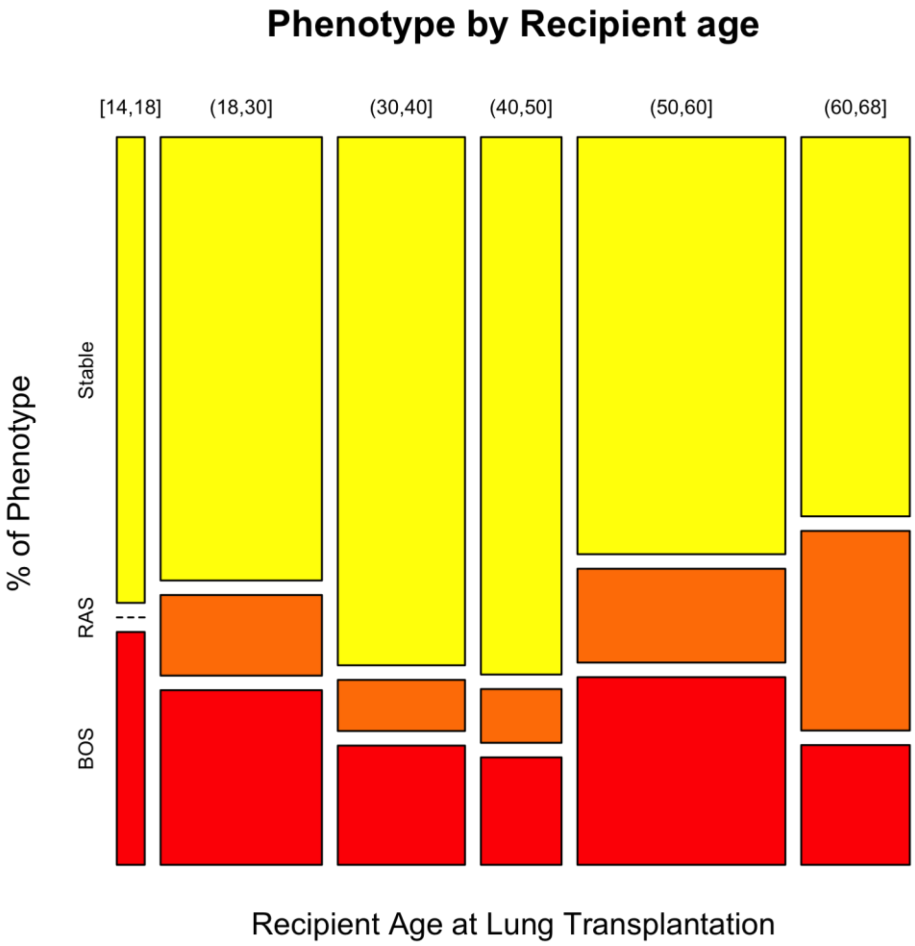
Phenotype by Recipient age
[14,18]
(18,30]
(30,40]
(40,50]
(50,60]
(60,68]
% of Phenotype
Stable
RAS
BOS
Recipient Age at Lung Transplantation
